# Expression of the plasma membrane citrate carrier (pmCiC) in human cancerous tissues—correlation with tumour aggressiveness

**DOI:** 10.3389/fcell.2024.1308135

**Published:** 2024-07-03

**Authors:** Barbara Schwertner, George Dahdal, Wolfgang Jagla, Luis Grossmann, Konstantin Drexler, Michael P. Krahn, Katja Evert, Mark Berneburg, Sebastian Haferkamp, Christine Ziegler, Eric K. Parkinson, Grit Zahn, Maria E. Mycielska, Andreas Gaumann

**Affiliations:** ^1^ Department of Dermatology, University Hospital Regensburg, Regensburg, Germany; ^2^ Institute of Pathology Kaufbeuren-Ravensburg, Kaufbeuren, Germany; ^3^ Department of Structural Biology, Institute of Biophysics and Physical Biochemistry, University of Regensburg, Regensburg, Germany; ^4^ Medical Cell Biology, Internal Medicine D, University Hospital Münster, Münster, Germany; ^5^ Institute of Pathology, University of Regensburg, Regensburg, Germany; ^6^ Centre for Oral Immunobiology and Regenerative Medicine, Institute of Dentistry, Barts and the London School of Medicine and Dentistry, Queen Mary University of London, London, United Kingdom; ^7^ Eternygen GmbH, Berlin, Germany

**Keywords:** biomarker, cancer, citrate, tumour microenvironment, plasma membrane citrate carrier (pmCiC)

## Abstract

We have recently shown that cancer cells of various origins take up extracellular citrate through the plasma membrane citrate carrier (pmCiC), a specific plasma membrane citrate transporter. Extracellular citrate is required to support cancer cell metabolism, in particular fatty acid synthesis, mitochondrial activity, protein synthesis and histone acetylation. In addition, cancer cells tend to acquire a metastatic phenotype in the presence of extracellular citrate. Our recent study also showed that cancer-associated stromal cells synthesise and release citrate and that this process is controlled by cancer cells. In the present study, we evaluated the expression of pmCiC, fibroblast activation protein-α (FAP) and the angiogenesis marker cluster of differentiation 31 (CD31) in human cancer tissues of different origins. In the cohort studied, we found no correlation between disease stage and the expression of FAP or CD31. However, we have identified a clear correlation between pmCiC expression in cancer cells and cancer-associated stroma with tumour stage. It can be concluded that pmCiC is increased in cancer cells and in cancer-supporting cells in the tumour microenvironment at the later stages of cancer development, particularly at the metastatic sites. Therefore, pmCiC expression has the potential to serve as a prognostic marker, although further studies are needed.

## 1 Introduction

Cancer cells require increased metabolism to meet the demands of rapid proliferation and metastatic activity (Warburg, 1925; Pascale et al., 2020). In order to do this, these cells need to employ specific metabolic pathways and supply them with appropriate metabolites (Romero-Garcia et al., 2011). One of the key substrates is citrate, which is considered the central metabolite in cancer cells (Icard et al., 2021). Citrate is a Krebs cycle intermediate and the primary substrate for fatty acid synthesis, a hallmark of cancer (Röhrig and Schulze, 2016). Citrate is normally synthesized intracellularly in the mitochondrial cycle in both, cancer and normal cells. However, cancer cells require excess citrate for cytosolic fatty acid synthesis, likely from the reverse Krebs cycle (Parkinson et al., 2021; Metallo et al., 2011). Cancer cells can also take up citrate from the extracellular space via the plasma membrane citrate transporter pmCiC [UniProt: D9HTE9, GenBank accession number: HM037273.1, NCBI Reference Sequences: NP_001243463.1 (protein), NM_001256534.2 (nucleotide)], which belongs to the SLC25 family (Mycielska et al., 2018). The pmCiC is a variant of the mitochondrial citrate carrier (mCiC) with a different start codon (Mazurek et al., 2010). The two transporters have individual first exons, but the rest of the sequence is identical (Mazurek et al., 2010). It already has been shown that pmCiC is expressed in human cancer tissues and that the transporter is increasingly present at the invasion front as well as at metastatic sites (Mycielska et al., 2018; Drexler et al., 2021). Cancer cells can take up extracellular citrate from the blood, but elevated levels are also found in some organs such as the liver, bone or brain [reviewed in Parkinson et al. (2021)]. Unfortunately, not much is known about citrate levels in specific human organs other than the prostate and brain, and even less is known about citrate levels in cancerous tissues (Parkinson et al., 2021). Our previous study showed that cancer cells deprived of extracellular citrate increased the expression of pmCiC in cancer-associated fibroblasts and this caused an increase in citrate export from these cells (Drexler et al., 2021). Recently, it has become clear that stromal support is crucial to promote cancer progression and sustain the process of metastasis (Xu et al., 2022; Lee et al., 2023). In particular, the tumour microenvironment has been shown to provide cancer cells with the necessary metabolites through the formation of new blood vessels as well as metabolic substrates released by cancer-associated cells (Eisenberg et al., 2020; Chen et al., 2021). The cancer-associated stroma has also been shown to release growth factors and modulate the immune response (Nishida, 2021; Chen et al., 2023). Tumour-associated macrophages play an important role in the cancer microenvironment. They are known to support tumour progression by establishing infrastructure and increasing the release of inflammatory factors (Zhou et al., 2019; Maller et al., 2021). Citrate uptake by cancer cells has been shown to support their metabolism (Mycielska et al., 2018). In particular, uptake of extracellular citrate decreased mitochondrial activity, reactive oxygen species synthesis and significantly reduced glucose uptake in human prostate cancer cells (Mycielska et al., 2018). Further studies showed that extracellular citrate enhanced the metastatic properties of cancer cells *in vitro* and *in vivo* (Drexler et al., 2022). Consistently, administration of gluconate, a specific blocker of pmCiC, reduced subcutaneous pancreatic tumour growth and metastatic spread in mice, as well as tumour growth and angiogenesis of Merkel cell carcinoma in the chorioallantoic membrane (CAM) assay (Drexler et al., 2021; Drexler et al., 2022). Daily administration of gluconate also reduced stromal transformation and increased tumour immune infiltration in mice (Drexler et al., 2022).

In the present study, we investigated whether pmCiC expression is associated with increased disease aggressiveness of cancer and can serve as a prognostic marker together with pmCiC expression in the stroma and blood vessels.

## 2 Materials and methods

### 2.1 Biopsies

The study included ninety-two patients with human gastrointestinal (20), lung (39), prostate (14) and urothelial (19) cancers. For each primary tumour (stage IV), the associated metastasis, if present, was also examined. This results in a maximum total number of 29 metastases. The tissues were retrospectively analyzed by immunohistochemistry. In some cases, it was not possible to perform and evaluate all the stainings for each biopsy because there was not enough material available. Accordingly, the number of stained sections varies depending on the antibody and the evaluation. One patient biopsy was excluded from the study because of missing information about the entity. The ethical commission of the Faculty for Medicine, University of Regensburg (14-101-0263), approved the study.

### 2.2 Immunohistochemistry (IHC)

Patient biopsies were fixed with buffered formalin and embedded in paraffin according to standard procedures (Kirstie Canene-Adams, 2013). Samples were cut into 2 µm thick slices. Slices were deparaffinized with xylene (Merck, Darmstadt, Germany) and rehydrated with ethanol (2 × 100% ethanol, 2 × 95% ethanol, 2 × 70% ethanol. Each step 5 min).

Immunohistochemical staining was performed with the primary antibodies anti-pmCiC (Mazurek et al., 2010; GenScript USA Inc., N-Terminal peptide of protein, Antigen Sequence CYDEVVKLLNKVWKTD, Host species: Rabbit, concentration 9.259 mg/mL, dilution for IHC 1:2200), anti-FAP (Abcam, ab240989, Anti-Fibroblast activation protein, alpha antibody [SP325] - BSA and Azide free, clone SP325, host species: rabbit, monoclonal; concentration 1.023 mg/mL, dilution for IHC 1:100, lot number GR3392926-3) and anti-CD31 [Zytomed Systems, MSK091-05 (0.5 mL concentrate), Mouse anti-CD31 (PECAM-1), clone JC70, host species: mouse, monoclonal, concentration 15.3 μg/mL, dilution for IHC 1:300, lot number A0479]. All primary antibodies used were from the same batch. The secondary antibodies used were included in the kits used for IHC (see below).

For pmCiC and FAP staining, the kit ZytoChem Plus HRP-Kit Rabbit (Zytomed/Biozol, Eching, Germany) was used, performing the protocol according to the manufacturer’s instructions. Epitope retrieval was performed with HIER Citrate Buffer pH6 (Zytomed) for anti-pmCiC and with HIER TRIS-EDTA Buffer pH 8.0 (Zytomed) for anti-FAP. Both buffers were heated to 90°C before the sections were steamed for 20 min. Sections were stained using AEC+ High-Sensitivity Substrate Chromogen Ready-to-Use (Dako/Agilent Technologies, Hamburg, Germany) and counterstained using hematoxylin (Carl Roth, Karlsruhe, Germany).

For CD31 staining, the kit ZytoChem Plus (AP) Polymer Kit (Zytomed/Biozol) was used, performing the protocol according to the manufacturer’s instructions. After the retrieval step sections were rinsed in cold water and PBS buffer. Then primary antibody was applied for 45 min at room temperature (RT). After a washing step, blocking buffer was applied for 30 min at RT. Then another washing step was done before antibody detection was induced applying the AP Polymer for 30 min at RT. Finally, sections were stained with the chromogen substrate alkaline phosphatase for 30 min at RT and counterstained with hematoxylin (Carl Roth).

### 2.3 Evaluation of immunohistochemical staining

All sections were scanned using a slide scanner (Pannoramic Scan from 3DHISTECHTM). Scanning was performed with a × 20 objective (Micrometer/pixel X: 0.242535; Micrometer/pixel Y: 0.242647). The scans were analyzed on the computer using the free software “CaseViewer” from 3DHistech (https://www.3dhistech.com/solutions/caseviewer/). Complete sections were analysed. All samples were scored blindly.

For the CD31 analysis, the areas of highest vascular density (hot spots) were identified on the entire sections by an experienced pathologist and up to five of these hot spots per section were then analysed/quantified. Snapshots were taken at virtual ×20 magnification (corresponding to the ×20 magnification on the microscope) for evaluation. Positive vessels were counted manually according to the international consensus for the evaluation of angiogenesis (Vermeulen et al., 2002). For each CD31 staining, five different sections were evaluated on the microscope at ×20 magnification using the hot spot method. The mean value was then calculated (from the up to five hot spots), providing the CD31 value for further analyses. Staining of pmCiC in blood vessels was analyzed by defining no pmCiC staining in vessels as 0 and detectable staining in vessels as 1.

A semi-quantitative assessment method called “histochemical score” (H-score) was used to evaluate the pmCiC and FAP stainings. We quantified the intensity and the extent of staining within tissue samples. Staining intensity was graded on a scale of 0–3, where 0 indicates no staining, 1 indicates weak staining, 2 indicates moderate staining, and 3 indicates strong staining. The extent of staining was evaluated by determining the percentage of positively stained cells within the sample, represented as a percentage ranging from 0% to 100% (in increments of 10). Intensity of staining and percentage were multiplied to generate the score of 300. A score 300 equal to 300 represents the highest possible score. An experienced pathologist carried out the IHC evaluation.

### 2.4 Statistics

Statistical analyses were performed using GraphPad Prism v. 8.4.2 (GraphPad Software Inc., San Diego, CA, United States). The *p* values for comparing groups were calculated using the Mann-Whitney test. Box plots show median and interquartile ranges in addition to minimum and maximum values. The line in the middle of the box represents the median. Correlations were evaluated using the Spearman’s rank correlation coefficient analysis. Two-tailed *p*-values ≤0.05 were considered statistically significant. A simple linear regression line has been included in the figures showing correlations to provide an additional visual aid to help understand the data. No statistical conclusions can be drawn from these linear regression lines.

## 3 Results

### 3.1 Expression of pmCiC, CD31, and FAP in primary tumors *versus* metastasis and their correlation with different tumor stages

**FIGURE 1:**
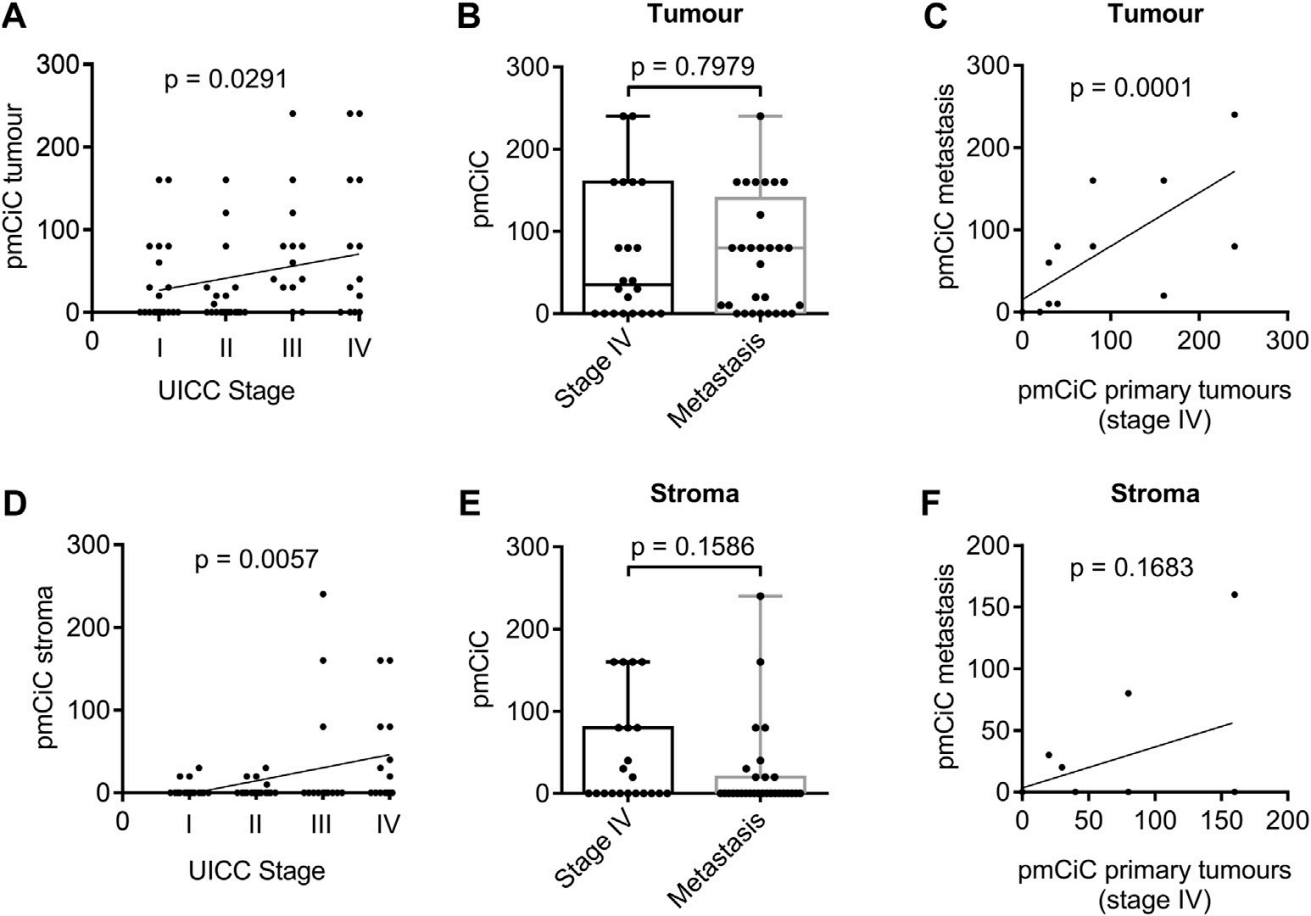
Expression of pmCiC in patient biopsies analysed by immunohistochemistry. **(A)** Spearman’s rank correlation coefficient analysis of pmCiC expression in cancer cells and stage. N = 75 (stage I: 20, stage II: 20, stage III: 13, stage IV: 22). **(B)** Box plot analysis of pmCiC expression in primary tumours of stage IV compared with metastasis. N = 51 (22 stage IV tumours, 29 metastasis). **(C)** Spearman’s rank correlation coefficient analysis of pmCiC in primary tumours stage IV and the corresponding metastases. **(D)** Spearman’s rank correlation coefficient analysis of pmCiC expression in the stroma surrounding cancer cells and stage. N = 73 (stage I: 20, stage II: 19, stage III: 13, stage IV: 21). **(E)** Box plot analysis of pmCiC expression in the stroma of primary tumours of stage IV compared with metastasis. N = 50 (21 stage IV tumours, 29 metastasis). **(F)** Spearman’s rank correlation coefficient analysis of pmCiC in the stroma of primary tumours stage IV and the corresponding metastases. Box plots show median and interquartile ranges in addition to minimum and maximum values. The line in the middle of the box represents the median. *p* values for box plots were calculated using the Mann-Whitney test. pmCiC expression is presented as Score 300.

We have recently found that pmCiC expression may play a role in the process of metastasis (Drexler et al., 2021), and therefore we wanted to investigate whether the level of pmCiC expression in cancer cells correlates with the tumour stage in human tissues. Indeed, pmCiC expression in tumour cells increased steadily with tumour stage (Figure 1A). With 45% of stage I samples, 40% of stage II samples, 84.6% of stage III samples, and 63.6% of stage IV samples showed pmCiC expression levels above zero. On the other hand, there was no difference in the expression of pmCiC between stage IV of primary tumours and metastatic tissues (Figure 1B). In fact, the distribution of pmCiC expression levels was very similar in both groups. Consistently, there was a clear correlation between the expression of pmCiC in stage IV primary tumours and the expression of pmCiC at the corresponding metastatic sites (Figure 1C). However, the number of tissues expressing pmCiC in cancer cells was higher in metastasis than in primary tissues at stage IV (63.6% and 72.4%, respectively).

Our recent study showed that citrate can be provided to cancer cells by the cancer-associated stroma and released from benign cells via pmCiC (Mycielska et al., 2018; Drexler et al., 2021). Furthermore, extracellular citrate has been shown to induce an invasive or colonising phenotype in cancer cells, so this metabolite exchange may be particularly important in supporting metastatic activity and the process of colonisation of distant organs. Correlating pmCiC expression in the stroma of the primary tumours with the tumour stage, showed an even stronger association than in the cancer cells themselves. The pmCiC expression in the stroma increased steadily with tumour stage (Figure 1D). This is consistent with stroma transformation and citrate supply being a critical element in the development of metastatic tumours. The results of the study indicate that 15% of stage I samples, 21.1% of stage II samples, 23.1% of stage III samples, and 47.6% of stage IV samples showed pmCiC expression levels above zero. Similar to pmCiC in cancer cells, there was no statistically significant difference between pmCiC expression in the stroma of cancer cells in primary stage IV tumour sites and in metastases (Figure 1E). We did not observe a correlation between pmCiC expressed in the stroma of stage IV tissues and metastasis (Figure 1F), however this could be due to the small number of tissues available for analysis. Moreover, there was a small decline in the number of metastatic tissues expressing pmCiC compared to the number of tissues expressing pmCiC in the stroma at stage IV, 47.6% and 31%, respectively.

**FIGURE 2:**
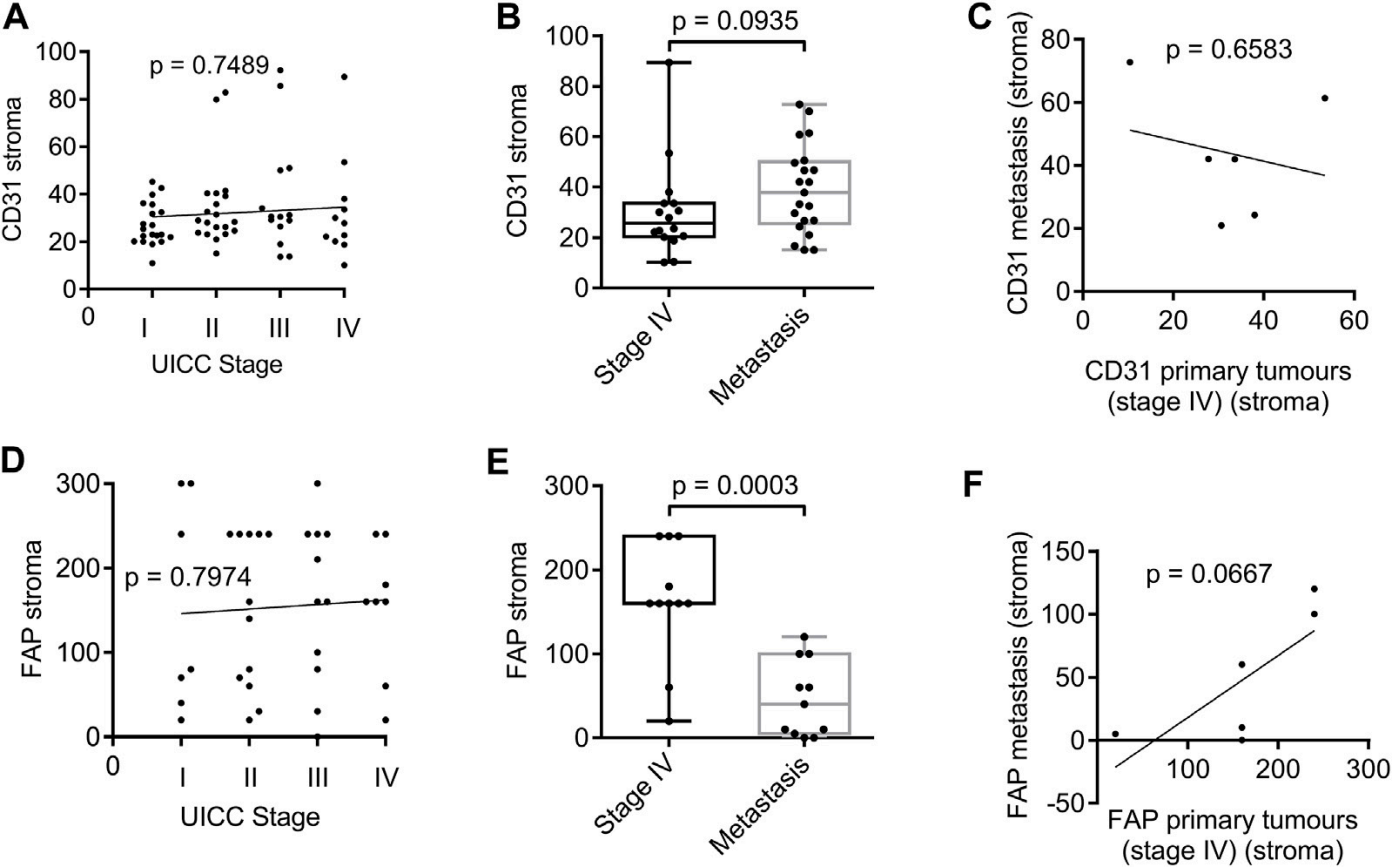
Expression of CD31 and FAP in the stroma surrounding tumour tissue of patient biopsies analysed by immunohistochemistry. **(A)** Spearman’s rank correlation coefficient analysis of CD31 expression and stage. N = 70 (stage I: 20, stage II: 20, stage III: 14, stage IV: 16). **(B)** Box plot analysis of CD31 expression in the stroma of primary tumours of stage IV compared with metastasis. N = 37 (16 stage IV tumours, 21 metastasis). **(C)** Spearman’s rank correlation coefficient analysis of CD31 in the stroma of primary tumours stage IV and the corresponding metastases. **(D)** Spearman’s rank correlation coefficient analysis of FAP expression and stage. N = 41 (stage I: 7, stage II: 12, stage III: 11, stage IV: 11). **(E)** Box plot analysis of FAP expression in the stroma of primary tumours of stage IV compared with metastasis. N = 22 (11 stage IV tumours, 11 metastasis). **(F)** Spearman’s rank correlation coefficient analysis of FAP in the stroma of primary tumours stage IV and the corresponding metastases. *p* values for box plots were calculated using the Mann-Whitney test. Box plots show median and interquartile ranges in addition to minimum and maximum values. The line in the middle of the box represents the median. Expression of CD31 is presented as number of positive vessels (mean value). FAP expression is presented as Score 300.

For successful disease progression and metastasis, cancer cells require support from the surrounding tissue, usually through the formation of new blood vessels and activation of the stroma. We used CD31, a marker of endothelial cells, and FAP, expressed by activated fibroblasts, to study angiogenesis and stroma formation in human cancer tissues. Interestingly, markers of angiogenesis and stromal activation showed no correlation with tumour stage when analysing the expression of CD31 (Figure 2A) or FAP (Figure 2D). In fact, the expression of CD31 and FAP in human cancer tissues showed a fairly even distribution across the different tumour stages, suggesting that their expression is important for tumour development, but does not necessarily need to increase at later stages of tumour progression. There was also no difference between CD31 expression levels in primary stage IV cancer tissues compared to metastases (Figure 2B). Thus, there was no correlation between CD31 expression in the primary tumour and the corresponding metastases (Figure 2C). However, noticeably, FAP expression was clearly reduced when comparing metastatic tissues with stage IV primary tumour sites (Figure 2E). No correlation of FAP expression was observed between metastatic tissues and corresponding stage IV primary tumours (Figure 2F).

### 3.2 Correlation of the pmCiC expression in the stroma *versus* cancer cells

**FIGURE 3:**
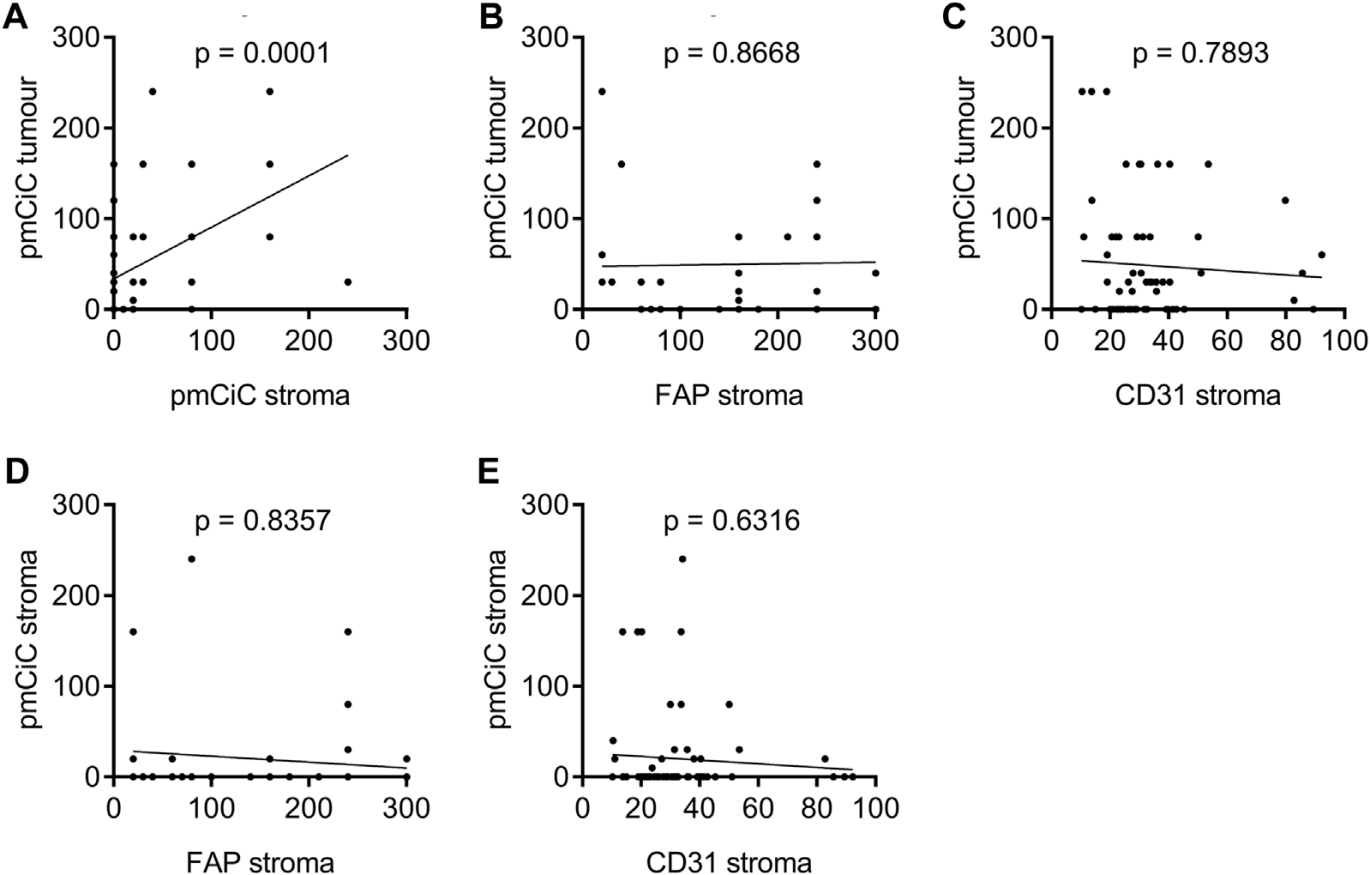
Spearman’s rank correlation coefficient analysis of pmCiC expression in cancer cells of primary tumours or in the surrounding tumour stroma, and FAP and CD31 in the surrounding tumour stroma analysed by immunohistochemistry. Correlation of pmCiC expression in cancer cells in tumour tissue and **(A)** pmCiC expression in the surrounding tumour stroma (N = 73), and **(B)** FAP expression (N = 40), and **(C)** CD31 expression (N = 68). Correlation of pmCiC expression in the primary tumour stroma and **(D)** FAP expression (N = 39), and **(E)** CD31 expression (N = 67). Expression of pmCiC and FAP is presented as Score 300. Expression of CD31 is presented as number of positive vessels (mean value).

Our recent study showed that cancer cells control the release of citrate from their local environment and that the level of citrate release depends on the metabolic needs of the cancer (Drexler et al., 2021). That is why we studied whether the expression of pmCiC in cancer cells correlates with pmCiC in the stroma. Indeed, we observed a correlation between these two markers in primary tumours between pmCiC in cancer cells and pmCiC in the stroma (Figure 3A). These data could confirm that the degree of transformation of the cancer-associated stroma depends on the specific metabolic needs of the cancer cells. In this case, the increased expression of pmCiC in cancer cells suggests a higher demand for extracellular citrate by cancer cells. This correlates with increased pmCiC expression in the surrounding stroma, which is consistent with increased release of this metabolite. This correlation was not observed when the level of expression of either FAP (Figure 3B) or CD31 (Figure 3C) in the stroma was examined in relation to pmCiC in tumour cells.

To determine whether the increased activity of the cancer-associated stroma, as indicated by increased citrate release, correlates with FAP and CD31, known markers of stromal formation, we correlated the expression of FAP *versus* pmCiC in the stroma (Figure 3D) and CD31 *versus* pmCiC expression in the stroma (Figure 3E). No correlation was observed with any of the markers tested, suggesting that the expression of pmCiC in the stroma is independent of the classical markers of stromal activation and angiogenesis.

### 3.3 Expression of pmCiC in blood vessels

**FIGURE 4:**
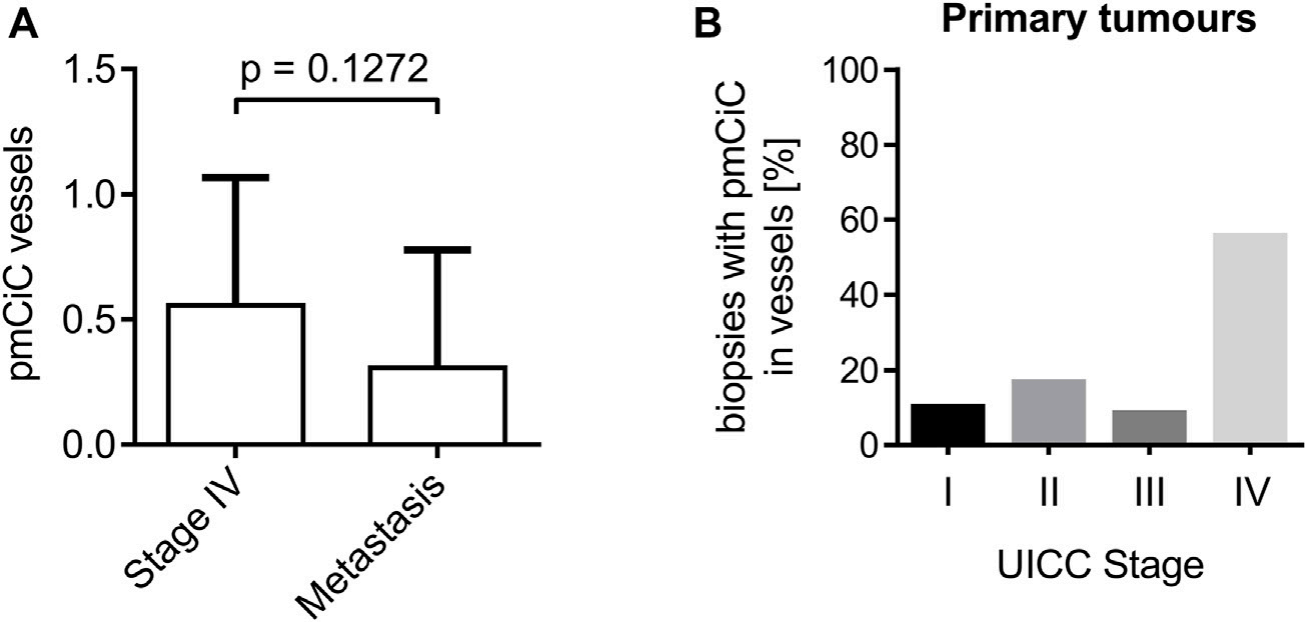
Analysis of pmCiC expression in blood vessels. **(A)** Analysis of pmCiC expression in vessels of stage IV tumours compared with metastasis. N = 44 (18 stage IV tumours, 26 metastasis). The *p* value was calculated using the Mann-Whitney test. The graph shows the mean with standard deviation. **(B)** Percentage of biopsies with pmCiC staining in blood vessels subdivided by tumour stage.

We have previously observed a certain level of pmCiC expression in some of the blood vessels present in human cancer tissue (Drexler et al., 2021; Parkinson et al., 2021). The analysis performed here showed that the presence of pmCiC-stained vessels is increased at later stages of cancer development. There was no statistical significance between pmCiC expression of blood vessels in primary stage IV tumour sites and metastasis (Figure 4A). A steep increase in the occurrence of vessels with pmCiC was observed in stage IV of cancer (Figure 4B).

### 3.4 Illustration of the data

**FIGURE 5:**
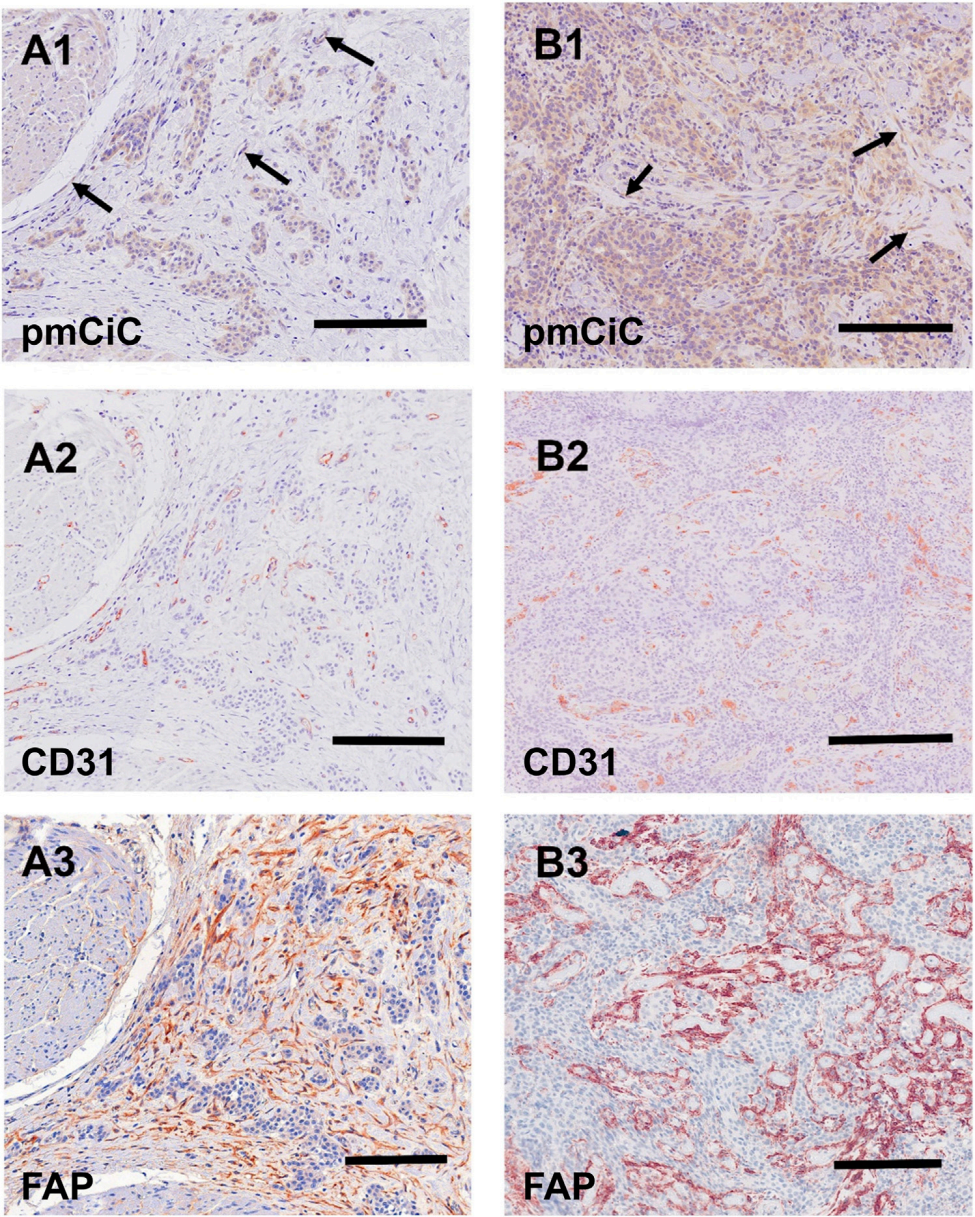
Immunostaining of two urothelial carcinomas. **(A1–A3)** (scale bar 100 µm) shows a primary muscle-infiltrating urothelial transitional cell carcinoma with positivity for pmCiC in cancer cells **(A1)** and endothelial cells **(A1; →)**. Serial sections show numerous vessels stained for CD31 **(A2)** as well as strong stromal reaction with FAP **(A3)**. **(B1–B3)** (scale bar 100 µm) displays another poorly differentiated urothelial cancer with prominent expression of pmCiC in cancer cells **(B1)** but also in some vessels **(B1; →)**. Serial section was stained with CD31 (**(B2)**; scale bar 150 µm) to highlight the tumour-associated vasculature. In **(B3)** there is a strong stromal reaction stained by FAP staining. All samples were counterstained with hematoxylin.

**FIGURE 6:**
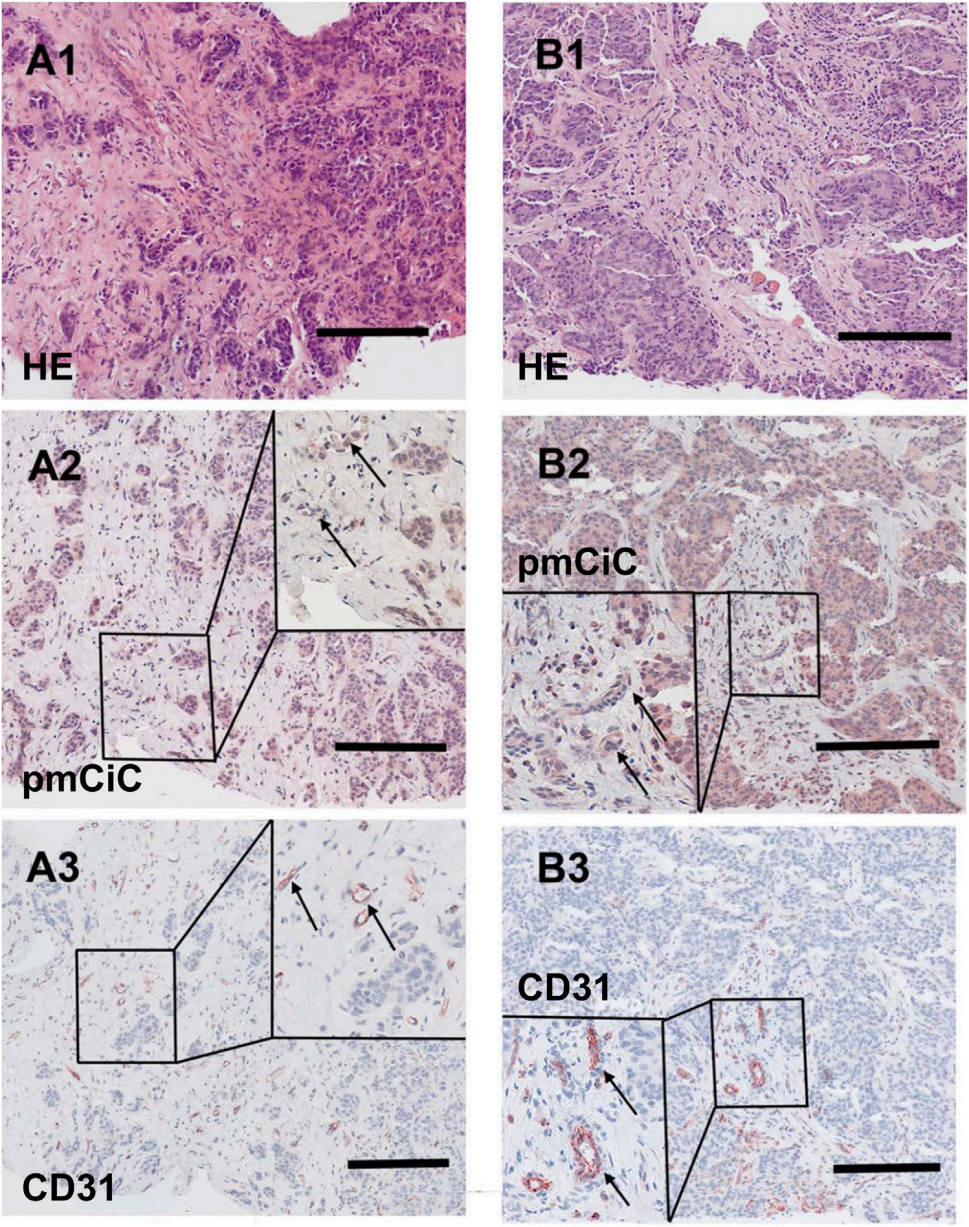
Histological staining of a liver metastasis of a ductal breast cancer and a liver metastasis of a prostate cancer. [**(A1–A3)**; scale bar 100 µm] Liver metastasis of a ductal breast cancer [**(A1)**; HE] shows little nest of atypical infiltrating cancer cells with a desmoplastic stroma expressing pmCiC in tumour cells **(A2)** as well as some associated small vessels (inset; →; magnification 3 - fold). In **(A3)** serial section of the metastasis shows tumour associated vessels stained for CD31 (inset; →; magnification 3 - fold). [**(B1–B3)**; scale bar 100 µm] Liver metastasis of a prostate cancer [**(B1)**; HE] displaying a pseudoglandulär atypical cancer with a desmoplastic stroma expressing pmCiC in tumour cells **(B2)** and few tumour-associated vessels (inset; →; magnification 3 - fold). In **(B3)** a serial section of the specimen was stained with CD31 to highlight the tumour associated vessels (inset; →; magnification 3 - fold). Images provide an overview and details at higher magnification (inserts; magnification 3 - fold); corresponding size bars are included. Tissues shown in **(A2, A3, B2, B3)** were counterstained with hematoxylin.

Figures 5, 6 summarise the analysis presented above. We chose to compare staining with different markers on consecutive sections of the same tissue. Figure 5 shows two different poorly differentiated urothelial tumours with prominent pmCiC staining in the cancer cells, but also in some vessels and the surrounding stroma. In contrast, Figure 6 shows liver metastases from ductal breast cancer (A1-A3) and prostate cancer (B1-B3).

It can be deduced that pmCiC expression is similarly high in poorly differentiated and metastatic cells. Importantly, higher expression of pmCiC in cancer cells correlates with increased levels of stromal cells also expressing pmCiC, particularly in Figure 5B1. This could be due to increased metastatic activity of the cancer cells or a lack of sufficient citrate at this site. Interestingly, although the stroma shown in Figure 6 is highly desmoplastic, the level of pmCiC expression in these cells is lower compared to primary urothelial tumours. This may be because citrate is abundant in the liver [reviewed in Parkinson et al. (2021)] and cancer cells have less need for this substrate. Figures 5, 6 show striking differences in the number and size of blood vessels stained with pmCiC and CD31, suggesting that pmCiC is an earlier marker of angiogenesis than CD31.

## 4 Discussion

For the purpose of the present study, we have combined human cancer tissues of different origins and evaluated the expression of pmCiC in cancer and stromal cells to check whether extracellular citrate can be considered as a necessary factor indicating the aggressiveness of the disease. We also put forward our hypothesis that the expression levels of pmCiC in cancer cells *versus* stroma would correlate with the amount of citrate uptake *versus* release, respectively. To assess other parameters of the tumour microenvironment, we also used the markers CD31 (Sharma et al., 2013; Bösmüller et al., 2018) and FAP (Ma et al., 2017; Coto-Llerena et al., 2020), which should reflect angiogenesis and fibroblast activation in the tissues studied. It should be noted that the limited number of tissues and the analysis performed without distinguishing for the tumour type might have affected some of the parameters by increasing the variability of the data.

Our study shows for the first time that pmCiC expression in cancer cells in human tissues correlates with tumour stage and is significantly increased at advanced stages of tumour development and metastatic sites, irrespective of tumour origin. This observation supports our recent data suggesting a role for extracellular citrate uptake in the acquisition of a more invasive (epithelial-mesenchymal transition (EMT)) or colonising (mesenchymal-epithelial transition (MET)) character of cancer cells, depending on the duration of citrate presence in the media (Drexler et al., 2021). Therefore, the expression of pmCiC in cancer cells could indicate an increased metastatic potential. In line with this hypothesis, pmCiC expression was less frequent in early stages of tumour development where its expression could indicate an increased likelihood of metastasis formation.

Extracellular citrate is normally present at stable concentrations in the blood and this is one of the sources of citrate for cancer cells, particularly those close to blood vessels. However, this source may not be sufficient as the tumour grows. We have shown that the tumour microenvironment is an additional source of citrate and that the amount of citrate released by cancer-associated fibroblasts depends on the availability of extracellular citrate to cancer cells. Therefore, citrate plays an important role in the communication between cancer cells and the surrounding stroma. Increased or decreased intracellular levels of citrate can be detrimental to cancer cells (Icard et al., 2019; Haferkamp et al., 2020); therefore, this controlled enrichment of the cancer microenvironment with citrate may represent a very interesting and not yet fully understood regulatory mechanism specific to cancer development (Jordan et al., 2022). Not surprisingly, our data from human cancer tissues show a correlation between pmCiC expression in cancer cells and pmCiC expression in the tumour environment, which may indicate a balance between citrate uptake and release and active stromal support of the disease progression. However, due to different levels of extracellular citrate and proximity to blood vessels, the expression of pmCiC in the stroma of primary tumours may also vary from organ to organ. For these reasons, it is likely that the simultaneous assessment of pmCiC expression in cancer cells and their microenvironment as well as in blood vessels could be a good prognostic marker for disease progression, rather than each of these elements separately. This aspect requires further and more detailed studies.

Some recent studies have shown the involvement of another sodium-dependent citrate transporter, NaCT (encoded by *SLC13A5*), in the metabolism of liver cancer cells (Li et al., 2017; Kumar et al., 2021). Specifically, citrate uptake in liver cancer cells reduced reductive carboxylation by providing citrate to ATP citrate lyase (ACYL) (Kumar et al., 2021). However, in liver cancer cells, this effect of extracellular citrate on cancer metabolism was particularly pronounced under conditions of reduced extracellular glucose and glutamine. On the other hand, our published study suggests that even under non-starving conditions, extracellular citrate uptake via pmCiC has a significant impact on fatty acid synthesis (Mycielska et al., 2018; Drexler et al., 2021). SLC13A5 also has a very restricted expression pattern. RNA expression data show expression only in liver cancer (Human Protein Atlas, 2023). Our very preliminary staining of human liver tissue may suggest that pmCiC may also be expressed in non-differentiated liver cancer cells (data not shown). Therefore, it remains crucial to investigate in the future which citrate transporters (or both) support metastasis.

There are also several groups suggesting that increasing extracellular citrate levels results in increased citrate uptake by cancer cells, leading to their death (Lu et al., 2011; Icard et al., 2023). In any case, it is suggested that extracellular citrate and plasma membrane citrate transporters play a significant role either in supporting cancer progression (at physiological levels) or with the potential to destroy cancer (when applied at very high levels).

We also found no correlation between CD31 or FAP expression and tumour stage. There is no agreement regarding the use of CD31 in the field of cancer prognosis. Some studies concluding CD31 to be a prognostic marker (Sandlund et al., 2007; Schmidt et al., 2017; Schlüter et al., 2018), but others failing to confirm this correlation (Rask et al., 2019). Finally, some other studies have found an inverse correlation between CD31 expression and patient survival (Virman et al., 2015; Emmert et al., 2016). In addition, the lack of correlation between CD31 and tumour stage in the present study may be due to the fact that we analysed tumours of different origin together in one group. As different organs have different blood vessel densities, this could affect the overall assessment.

Although we did not find a correlation between CD31 and FAP expression and disease stage, there were increased levels of pmCiC present in the blood vessels of later stage cancers. CD31 is an endothelial cell marker (Bösmüller et al., 2018) and stains all blood vessels at any stage from early to full vascularization. In contrast, we found that pmCiC is only expressed in a small fraction of tiny blood vessels, most likely in the early formation stage, but not in well differentiated vessels. This would be consistent with increased citrate uptake by pmCiC at the earlier stage of vessel development. Citrate, as the primary substrate for fatty acid synthesis, could contribute to the formation and modification of the plasma membrane necessary for angiogenesis. Blocking fatty acid synthesis has already been shown to affect angiogenesis. In a murine stroke model, the use of cerulenin increased endothelial cell leakage, decreased transcellular electrical resistance and contributed to the breakdown of the blood-brain barrier (BBB) after stroke (Janssen et al., 2021). Knockdown of fatty acid synthase inhibited vessel sprouting by reducing cell proliferation (Bruning et al., 2018). Strikingly, most of the pmCiC-expressing blood vessels were found at stage IV of tumour development. It is possible that these vessels are involved in/facilitate the dissemination of metastatic cells, e.g., due to controlled fatty acid content or other special characteristics. Therefore, the expression of pmCiC in endothelial cells may be a specific feature of early cancer angiogenesis and a different factor to CD31, but this issue remains to be investigated.

The lack of correlation between CD31 and FAP expression in the stroma and cancer stage could be due to several reasons. Firstly, a limited number of tissues examined could certainly play a role in obtaining significant results. In addition, tumours of different origin have different requirements for stromal support (Parkinson et al., 2021). In this case, the combination of all tumour types in one group could further influence the results obtained. In this context, however, it is even more striking that the expression of pmCiC and its correlation with the stage of the tumour were independent of the origin of the tumour and could therefore represent a novel and widely applicable marker of tumour aggressiveness.

We have hypothesised that elevated citrate levels in organs such as the brain, bone or liver facilitate organ colonisation and are therefore the most common sites of secondary tumour growth (Parkinson et al., 2021). In this respect, the variability in the expression of pmCiC in cancer and peritumour tissues, as shown in the present study, could also be caused by different levels of extracellular citrate in the organs from which the tissues were obtained. It is interesting to note that the number of metastatic tissues stained with pmCiC in the stroma is lower than in the primary tumours at stage IV. This observation would be consistent with the fact that the most common organs in which distant metastasis occur, such as the liver, brain, bone or the lung, are rich in citrate (Parkinson et al., 2021). Citrate levels need to be tightly controlled by cancer cells, as its increase was shown to be detrimental to cancer. Since colonising cancer cells in an extracellular citrate-rich environment are less likely to demand additional citrate from the surrounding stroma, it is expected that pmCiC expression will be lower. However, the number of metastatic tissues expressing pmCiC in cancer cells compared to the primary tumours at stage IV increases suggesting an important role of extracellular citrate in organ colonisation.

## 5 Conclusion

In conclusion, the expression level of pmCiC in cancer cells from human cancer tissues seem to increase with tumour stage and is particularly elevated at metastatic sites. Importantly, we also found elevated levels of pmCiC in the tumour microenvironment, which may indicate the importance of extracellular citrate in the progression of metastatic disease. However, the exact role of citrate in metastatic progression remains to be elucidated and will be addressed in our next studies. On the other hand, we did not observe significant correlations of other stromal markers, CD31 and FAP, with tumour stage. It is possible that the expression of pmCiC in cancer cells and the supporting stroma may be an early event in the process of metastasis and organ colonization. It is worth noticing that we performed our study on human cancerous tissues of different origins. To our knowledge this is one of the first studies showing a common factor/feature correlating with tumour aggressiveness. Therefore, it should be further investigated in the context of a prognostic marker.

## Data Availability

The raw data supporting the conclusions of this article will be made available by the authors, without undue reservation.

## References

[B1] Bösmüller H. Pfefferle V. Bittar Z. Scheble V. Horger M. Sipos B. (2018). Microvessel density and angiogenesis in primary hepatic malignancies: differential expression of CD31 and VEGFR-2 in hepatocellular carcinoma and intrahepatic cholangiocarcinoma. Pathol. Res. Pract. 214, 1136–1141. 10.1016/j.prp.2018.06.011 29935812

[B2] Bruning U. Morales-Rodriguez F. Kalucka J. Goveia J. Taverna F. Queiroz K. C. S. (2018). Impairment of angiogenesis by fatty acid synthase inhibition involves mTOR malonylation. Cell. Metab. 28, 866–880. 10.1016/j.cmet.2018.07.019 30146486 PMC8057116

[B3] Chen Q. Y. Gao B. Tong D. Huang C. (2023). Crosstalk between extracellular vesicles and tumor-associated macrophage in the tumor microenvironment. Cancer Lett. 552, 215979. 10.1016/j.canlet.2022.215979 36306939

[B4] Chen Y. McAndrews K. M. Kalluri R. (2021). Clinical and therapeutic relevance of cancer-associated fibroblasts. Nat. Rev. Clin. Oncol. 18, 792–804. 10.1038/s41571-021-00546-5 34489603 PMC8791784

[B5] Coto-Llerena M. Ercan C. Kancherla V. Taha-Mehlitz S. Eppenberger-Castori S. Soysal S. D. (2020). High expression of FAP in colorectal cancer is associated with angiogenesis and immunoregulation processes. Front. Oncol. 10, 979. 10.3389/fonc.2020.00979 32733792 PMC7362758

[B6] Drexler K. Schmidt K. M. Jordan K. Federlin M. Milenkovic V. M. Liebisch G. (2021). Cancer-associated cells release citrate to support tumour metastatic progression. Life Sci. 4, e202000903. 10.26508/lsa.202000903 PMC7994318 33758075

[B7] Drexler K. Schwertner B. Haerteis S. Aung T. Berneburg M. Geissler E. K. (2022). The role of citrate homeostasis in Merkel cell carcinoma pathogenesis. Cancers (Basel) 14, 3425. 10.3390/cancers14143425 35884486 PMC9325124

[B8] Eisenberg L. Eisenberg-Bord M. Eisenberg-Lerner A. Sagi-Eisenberg R. (2020). Metabolic alterations in the tumor microenvironment and their role in oncogenesis. Cancer Lett. 484, 65–71. 10.1016/j.canlet.2020.04.016 32387442

[B9] Emmert A. Oellerich A. Füzesi L. Waldmann-Beushausen R. Bohnenberger H. Schöndube F. A. (2016). Prognostic significance of CD31 expression in patients with non-small-cell-lung cancer. ALC 05, 21–29. 10.4236/alc.2016.53003

[B10] Haferkamp S. Drexler K. Federlin M. Schlitt H. J. Berneburg M. Adamski J. (2020). Extracellular citrate fuels cancer cell metabolism and growth. Front. Cell. Dev. Biol. 8, 602476. 10.3389/fcell.2020.602476 33425906 PMC7793864

[B11] Human Protein Atlas (2023). Human protein Atlas. SCL13A5. Available at: https://www.proteinatlas.org/ENSG00000141485-SLC13A5/pathology (Accessed December 11, 2023).

[B12] Icard P. Alifano M. Simula L. (2023). The potential for citrate to reinforce epigenetic therapy by promoting apoptosis. Trends Endocrinol. Metbo 34, 586–589. 10.1016/j.tem.2023.07.002 37550099

[B13] Icard P. Coquerel A. Wu Z. Gligorov J. Fuks D. Fournel L. (2021). Understanding the central role of citrate in the metabolism of cancer cells and tumors: an update. Int. J. Mol. Sci. 22, 6587. 10.3390/ijms22126587 34205414 PMC8235534

[B14] Icard P. Wu Z. Alifano M. Fournel L. (2019). Gluconeogenesis of cancer cells is disrupted by citrate. Trends Cancer 5, 265–266. 10.1016/j.trecan.2019.03.002 31174837

[B15] Janssen L. Ai X. Zheng X. Wei W. Caglayan A. B. Kilic E. (2021). Inhibition of fatty acid synthesis aggravates brain injury, reduces blood-brain barrier integrity and impairs neurological recovery in a murine stroke model. Front. Cell. Neurosci. 15, 733973. 10.3389/fncel.2021.733973 34483846 PMC8415573

[B16] Jordan K. Stanton E. H. Milenkovic V. M. Federlin M. Drexler K. Buchalla W. (2022). Potential involvement of extracellular citrate in brain tumor progression. Curr. Mol. Med. 22, 506–513. 10.2174/1566524021666210302143802 33653247

[B17] Kirstie Canene-Adams K. (2013). Preparation of formalin-fixed paraffin-embedded tissue for immunohistochemistry. Methods Enzym. 533, 225–233. 10.1016/B978-0-12-420067-8.00015-5 24182927

[B18] Kumar A. Cordes T. Thalacker-Mercer A. E. Pajor A. M. Murphy A. N. Metallo C. M. (2021). NaCT/SLC13A5 facilitates citrate import and metabolism under nutrient-limited conditions. Cell. Rep. 36, 109701. 10.1016/j.celrep.2021.109701 34525352 PMC8500708

[B19] Lee T.-L. Chen T.-H. Kuo Y.-J. Lan H.-Y. Yang M.-H. Chu P.-Y. (2023). Tumor-associated tissue eosinophilia promotes angiogenesis and metastasis in head and neck squamous cell carcinoma. Neoplasia 35, 100855. 10.1016/j.neo.2022.100855 36410227 PMC9677212

[B20] Li Z. Li D. Choi E. Y. Lapidus R. Zhang L. Huang S.-M. (2017). Silencing of solute carrier family 13 member 5 disrupts energy homeostasis and inhibits proliferation of human hepatocarcinoma cells. J. Biol. Chem. 292, 13890–13901. 10.1074/jbc.M117.783860 28655760 PMC5566540

[B21] Lu Y. Zhang X. Zhang H. Lan J. Huang G. Varin E. (2011). Citrate induces apoptotic cell death: a promising way to treat gastric carcinoma? Anticancer Res. 31, 797–805. 21498699

[B22] Ma T. H. Gao C. C. Xie R. Yang X. Z. Dai W. J. Zhang J. L. (2017). Predictive values of FAP and HGF for tumor angiogenesis and metastasis in colorectal cancer. Neoplasma 64, 880–886. 10.4149/neo_2017_609 28895412

[B23] Maller O. Drain A. P. Barrett A. S. Borgquist S. Ruffell B. Zakharevich I. (2021). Tumour-associated macrophages drive stromal cell-dependent collagen crosslinking and stiffening to promote breast cancer aggression. Nat. Mat. 20, 548–559. 10.1038/s41563-020-00849-5 PMC8005404 33257795

[B24] Mazurek M. P. Prasad P. D. Gopal E. Fraser S. P. Bolt L. Rizaner N. (2010). Molecular origin of plasma membrane citrate transporter in human prostate epithelial cells. EMBO Rep. 11, 431–437. 10.1038/embor.2010.51 20448665 PMC2892322

[B25] Metallo C. M. Gameiro P. A. Bell E. L. Mattaini K. R. Yang J. Hiller K. (2011). Reductive glutamine metabolism by IDH1 mediates lipogenesis under hypoxia. Nature 481, 380–384. 10.1038/nature10602 22101433 PMC3710581

[B26] Mycielska M. E. Dettmer K. Rümmele P. Schmidt K. Prehn C. Milenkovic V. M. (2018). Extracellular citrate affects critical elements of cancer cell metabolism and supports cancer development *in vivo* . Cancer Res. 78, 2513–2523. 10.1158/0008-5472.CAN-17-2959 29510993

[B27] Nishida N. (2021). Role of oncogenic pathways on the cancer immunosuppressive microenvironment and its clinical implications in hepatocellular carcinoma. Cancers (Basel) 13, 3666. 10.3390/cancers13153666 34359568 PMC8345137

[B28] Parkinson E. K. Adamski J. Zahn G. Gaumann A. Flores-Borja F. Ziegler C. (2021). Extracellular citrate and metabolic adaptations of cancer cells. Cancer Metastasis Rev. 40, 1073–1091. 10.1007/s10555-021-10007-1 34932167 PMC8825388

[B29] Pascale R. M. Calvisi D. F. Simile M. M. Feo C. F. Feo F. (2020). The warburg effect 97 Years after its discovery. Cancers (Basel) 12, 2819. 10.3390/cancers12102819 33008042 PMC7599761

[B30] Rask L. Høgdall C. K. Kjaer S. K. Christensen L. Jensen A. Blaakaer J. (2019). Association of CD31 and p53 with survival of ovarian cancer patients. Anticancer Res. 39, 567–576. 10.21873/anticanres.13149 30711931

[B31] Röhrig F. Schulze A. (2016). The multifaceted roles of fatty acid synthesis in cancer. Nat. Rev. Cancer 16, 732–749. 10.1038/nrc.2016.89 27658529

[B32] Romero-Garcia S. Lopez-Gonzalez J. S. Báez-Viveros J. L. Aguilar-Cazares D. Prado-Garcia H. (2011). Tumor cell metabolism: an integral view. Cancer Biol. Ther. 12, 939–948. 10.4161/cbt.12.11.18140 22057267 PMC3280912

[B33] Sandlund J. Hedberg Y. Bergh A. Grankvist K. Ljungberg B. Rasmuson T. (2007). Evaluation of CD31 (PECAM-1) expression using tissue microarray in patients with renal cell carcinoma. Tumour Biol. 28, 158–164. 10.1159/000102980 17510564

[B34] Schlüter A. Weller P. Kanaan O. Nel I. Heusgen L. Höing B. (2018). CD31 and VEGF are prognostic biomarkers in early-stage, but not in late-stage, laryngeal squamous cell carcinoma. BMC Cancer 18, 272. 10.1186/s12885-018-4180-5 29523110 PMC5845191

[B35] Schmidt L. H. Brand C. Stucke-Ring J. Schliemann C. Kessler T. Harrach S. (2017). Potential therapeutic impact of CD13 expression in non-small cell lung cancer. PLoS One 12, e0177146. 10.1371/journal.pone.0177146 28604784 PMC5467809

[B36] Sharma B. Singh N. Gupta N. Lal P. Pande S. Chauhan S. (2013). Diagnostic modalities of precancerous and cancerous cervical lesions with special emphasis on CD31 angiogenesis factor as a marker. Pathol. Res. Int. 2013, 243168. 10.1155/2013/243168 PMC3791664 24167753

[B37] Vermeulen P. B. Gasparini G. Fox S. B. Colpaert C. Marson L. P. Gion M. (2002). Second international consensus on the methodology and criteria of evaluation of angiogenesis quantification in solid human tumours. Eur. J. Cancer 38, 1564–1579. 10.1016/s0959-8049(02)00094-1 12142044

[B38] Virman J. Bono P. Luukkaala T. Sunela K. Kujala P. Kellokumpu-Lehtinen P.-L. (2015). VEGFR3 and CD31 as prognostic factors in renal cell cancer. Anticancer Res. 35, 921–927. 25667475

[B39] Warburg O. (1925). The metabolism of carcinoma cells. J. Cancer Res. 9, 148–163. 10.1158/jcr.1925.148

[B40] Xu M. Zhang T. Xia R. Wei Y. Wei X. (2022). Targeting the tumor stroma for cancer therapy. Mol. Cancer 21, 208. 10.1186/s12943-022-01670-1 36324128 PMC9628074

[B41] Zhou J. Zheng S. Liu T. Liu Q. Chen Y. Ma R. (2019). Infiltrated M2 tumour-associated macrophages in the stroma promote metastasis and poor survival in oesophageal squamous cell carcinoma. Histol. Histopathol. 34, 563–572. 10.14670/HH-18-061 30417922

